# Understanding the Evolution of Multimorbidity: Evidences from the North West Adelaide Health Longitudinal Study (NWAHS)

**DOI:** 10.1371/journal.pone.0096291

**Published:** 2014-05-05

**Authors:** Guillaume Ruel, Jean-Frédéric Lévesque, Nigel Stocks, Caroline Sirois, Edeltraut Kroger, Robert J. Adams, Mariève Doucet, Anne W. Taylor

**Affiliations:** 1 Ministère de la Santé et des Services sociaux, Québec, Québec, Canada; 2 Institut National de Santé Publique du Québec, Québec, Québec, Canada; 3 Population Research and Outcome Studies, University of Adelaide, Adelaide, South Australia, Australia; 4 Bureau of Health Information, Chatswood, New South Wales, Australia; 5 Centre de recherche du Centre hospitalier Universitaire de Montréal, Université de Montréal, Montréal, Québec, Canada; 6 Discipline of General Practice, University of Adelaide, Adelaide, South Australia, Australia; 7 Département de science infirmière, Université du Québec à Rimouski, Rimouski, Québec, Canada; 8 Département de pharmacie, Université Laval, Québec, Québec, Canada; 9 Health Observatory, University of Adelaide, Adelaide, South Australia, Australia; 10 Faculté de Médecine, Université Laval, Québec, Québec, Canada; Universidad Peruana Cayetano Heredia, Peru

## Abstract

**Objective:**

The aim of this study is to describe the evolution of multimorbidity.

**Study Design and Setting:**

Data from 1854 South Australians who participated in the North West Adelaide longitudinal Health Study(NWAHS) was collected between baseline (2000–2002) and follow-up (2008–2010). Status for eight chronic diseases (CDs) was determined by biomedical measurement or self-report. Chronic disease (CD) mean age of occurrence and order of appearance was investigated.

**Results:**

The prevalence of multimorbidity increased from 32% to 64% during the 7.8±1.1 years of follow-up. The estimated mean age of onset of a new CD was significantly older for hypertension, cardiovascular disease (CVD) and chronic obstructive pulmonary disease (COPD) and younger for hypercholesterolemia, asthma and other mental problem. Hypercholesterolemia was more likely to develop as a first than as a subsequent CD (39%*vs*.16%, p<0.0001) while CVD (1%*vs*.5%, p<0.0001), diabetes (5%*vs*.11%, p<0.001) and COPD (6%*vs*.16%, p<0.0001) were less likely. The presence of mood disorders at baseline was associated with an increased risk of developing other mental disorders (36%*vs*.12%, p<0.0001), diabetes (18%*vs*.9%, p<0.01) and asthma (30%*vs*.21%, p<0.05).

**Conclusion:**

Longitudinal data could be used to study the evolution of multimorbidity and could provide information on CDs mean age of occurrence, order of appearance and impact on the development of future CDs.

## Introduction

Today, chronic diseases (CDs) are highly prevalent and constitute the leading cause of death in the world [1]. However, advances in the management of CDs has slowed the progression of diseases and delayed related death in developed countries. Such changes have led to an increase in multimorbidity [2], [3], [4], [5], mainly defined as the presence of two or more medical conditions in an individual [6]. In general, multimorbidity includes only CDand its prevalence increases with age [3], [7] and social deprivation [7], [8]. It has been shown to be associated with increased medical consultation, prescription, psychological distress, emergency utilization, hospital length of stay, and mortality rates [9], [10], [11], [12].

Multimorbidity is part of a continuum ranging from healthy status, development of a single CDand then progression to multimorbidity with the addition of a further CD. The term ‘evolution of multimorbidity’ could be used to describe this whole process. The knowledge retrieved from a study of evolution of multimorbidity has the potential to identify populations at high risk of developing multimorbidity as well as repartitioning CDs across this continuum. Few studies [13], [14], [15], [16] have examined multimorbidity from a longitudinal perspective. Those studies focused mostly on the impact of CDs on disability, functional decline and mortality [14] or its relationship with nutrition [16]. In addition to the strength of association between CDs at a specific time point (e.g. observed over expected ratio) [17], a longitudinal follow-up design is rarely used in the context of multimorbidity and could help understand causality [15]. To the best of our knowledge, no study has used a longitudinal study design to investigate the evolution of multimorbidity. This study aims to characterize evolution of multimorbidity by 1) characterizing the magnitude of the evolution of the prevalence of multimorbidity through time, 2) determining the order in which CDs appear and 3) identifying CDs present at baseline which are associated with an increased risk of developing other CDs.

## Methods

### Study design

The North West Adelaide Health Longitudinal Study (NWAHS) is a representative population sample of people aged 18 years or older living in the north western suburbs of Adelaide, South Australia (regional population 0.6 million) and covers a broad range of socioeconomic areas. The study's purpose is to investigate the prevalence of chronic conditions and associated health-related risk factors, and to monitor progression of diseases over time in order to help plan health care provision. The sample is representative of the community profile of Adelaide [18], [19], [20] and has been described previously in detail [18], [19]. Briefly, between 2000 and 2002, 4060 adults completed a telephone interview, self-completed questionnaire and clinic biomedical assessment (including blood sample). This represents 50% of the people invited to take part in the study. Persons aged ≥18 years from households selected at random from the electronic white pages directory were eligible. Respondents completed a self-report questionnaire related to health status and additional demographic details and underwent clinical assessment, including measurement of blood pressure, height, weight, fasting glucose and lipid levels. Stage 2 (2004 to 2006) and Stage 3 (2008 to 2010) included a Computer Assisted Telephone Interview (CATI), a self completed questionnaire and a biomedical examination at a clinic. For Stage 2, 3564 of the participants were interviewed and/or completed a questionnaire and 81.0% of the eligible sample (n = 3205) attended a clinic appointment. For Stage 3, 2871 of the participants were interviewed and/or completed a questionnaire and 2487 individuals attended clinics. Overall, complete clinical and self reports information at each phase and for every CD was available for 1854 individuals (46% of the baseline sample). In order to maximize follow-up duration, the data from stage 1 and 3 were used, but data from stage 2 also contributed to establishing CD status at stage 3. Stage 1 and 3 are considered as baseline and follow-up throughout this article. A recently published paper by our group directly addresses the representativeness of the stage 3 cohort [21]. Briefly, it was found that compared to the census, and a sample of the population surveyed by CATI over the same period, those in the stage 3 of NWAHS were older, more likely to have a trade or certificate, to be employed, to be a non-smoker and to have a higher income. No significant differences were found in gender proportion and alcohol consumption. Requests for de-identified data can be sent to the Chief investigator (anne.taylor@adelaide.edu.au) or the study co-ordinator (Janet.grant@adelaide.edu.au). The study was approved by institutional ethics committees of the North West Adelaide Health Service, and all subjects gave written informed consent.

### Physical and Socio-demographic characteristics

Height was measured to the nearest 0.5 centimetres using a stadiometer, and weight to the nearest 0.1 kilogram in light clothing and without shoes using standard digital scales. Body mass index (BMI) was calculated as weight (kg)/height (m)^2^. Information on socio-demographic and other lifestyle factors were also collected (Table 1).

**Table 1:** Physical and socio-demographic characteristics of the 1854 participants to the North West Adelaide Health Study at baseline (2002).

Variable	N (%)
Age (mean ± SD, years old)	50±14
**Age groups**	
18–40	488 (26)
41–50	467 (25)
51–60	461 (25)
61–70	282 (15)
71 and over	156 (8)
**Sex**	
Male	882 (48)
Female	972 (52)
**Education**	
Secondary	768 (41)
Trade/Apprenticeship/certificate/Diploma	804 (43)
Bachelor degree or higher	241 (13)
Do not know/refused	41 (2)
**Occupation**	
Full time employed	782 (42)
Part time/casual employment	341 (18)
Unemployed	50 (3)
Home duties	235 (13)
Retired	364 (20)
Student	30 (2)
Other/not stated	52 (3)
**Annual Income**	
Up to $ 12,000	192 (10)
$ 12,001–$ 20,000	235 (13)
$ 20,001–$ 40,000	485 (26)
$ 40,001–$ 60,000	419 (23)
$ 60,001–$ 80,000	223 (12)
Over $ 80,000	228 (12)
Do not know/refused	72 (4)
**Do you receive pension or benefits from social security?**	
Yes	584 (32)
No	1251 (68)
Do not know/not stated	19 (1)
**Marital status**	
Married/living with partner	1237 (67)
Separated/divorced	272 (15)
Widowed	130 (7)
Never married	207 (11)
Not stated	8 (0)
**Family structure (from phase 2)**	
A family with a child	562 (30)
A step or blended family	82 (4)
A sole parent family	82 (4)
Shared care parenting	33 (2)
Adult living alone	341 (18)
Adult living with partner and with no child	582 (31)
Related adults living together	125 (7)
Unrelated adult living together	24 (1)
Other/Refused	23 (1)

### Chronic disease status assessments

CD status was determined for the following eight conditions: asthma, cardiovascular disease and stroke (CVD), chronic obstructive pulmonary disease (COPD), diabetes, mood and anxiety disorders, any other mental disorders, hypercholesterolemia and hypertension. Asthma, COPD, diabetes, hypercholesterolemia and hypertension were determined at the clinic visit. A fasting blood test was performed to determine total cholesterol and glucose levels (FPG). A person was deemed to have diabetes if they self-reported having ever been told by a doctor they had the condition, self-reported utilisation of glucose lowering drugs or by measurement at the clinic (FPG≥7.0 mmol/L) [22], [23]. High cholesterol was defined as total blood cholesterol ≥5.5 mmol/L or self report of cholesterol lowering drugs [24]. Two blood pressure measurements were taken five to ten minutes apart using a standard, calibrated blood pressure sphygmomanometer, while the participant was relaxed and seated. The average of these two recorded measures was used in the analyses. High blood pressure was defined as systolic blood pressure ≥140 mm Hg and/or diastolic blood pressure ≥90 mm Hg or the self-reported use of drugs to lower blood pressure. Participants were tested for asthma and COPD at the clinic visit by spirometry before and after the use of a beta agonist. A positive COPD case was present if FEV1/FVC<70% and for asthma if FEV1≥12% and >200 ml or absolute change greater or equal to 400 ml from pre salbutamol to post salbutamol [25], [26]. Status for asthma was determined using clinical and self-report while COPD was mainly based on clinical measurement as previously described [25], [26]. Participants self-reported if they had ever been told by a doctor they had CVD (heart attack, stroke, angina) or a mental health condition (anxiety, depression or other mental health problem). Mental health condition was then split between mood and anxiety disorders and other mental disorders. An individual having a diagnosis for a CD was considered as having it for every phase afterwards.

### Statistical analyses

Mean incident age of onset of a CD developed during the follow-up period was calculated based on age at the time of follow-up. Mean incident age of onset of a CD was compared with those that developed another CD by using independent sample student *t* test. For the population that developed a CD, multinomial logistic regression (MLR) was used to determine significant differences in the count and individual proportion of CDs developed between those who were healthy or had at least one CD at baseline. Among those with a CD at baseline, MLR was also used to determine significant differences in the count and proportions of those with CDs between who developed CDs or not. Difference in CDs developed by the subgroups with asthma or mood and anxiety disorders at baseline was determined using MLR. The control group consisted of those who developed another CD. All MLR analyses were undertaken for crude; adjusted by age and sex; and adjusted for age, sex, BMI status, marital status, working status, annual income and education. For all the MLR analyses, unadjusted odds ratios were also determined. Unless stated otherwise, (95%) confidence intervals are presented in the figures and standard deviation in the text while the *p* value found in the text is adjusted for both age and sex. All analyses were carried out using SPSS version 17.0 (SPSS, Inc., Chicago, IL, USA) and a p<0.05 was considered significant.

## Results

### Description of the cohort

Baseline physical and socio-demographic characteristics of the cohort are described in **Table 1**. Briefly, the cohort (mean age ± standard deviation at baseline: 50±14 years old) included slightly more females (52%), people working (60%) and married (67%). The mean follow-up period of the 1854 individuals included in the cohort was 7.8±1.1 years.

### Chronic diseases and multimorbidity status

CD status throughout the study is presented in **Table 2**. At baseline, among the 1854 participants, 1299 (70%) had at least one CD at baseline. The prevalence of multimorbidity doubled from baseline (32%; 95%CI: 30–34%) to follow-up (64%; 62–66%). Accordingly, the mean number of CDs per person increased from 1.12±0.99 to 2.09±1.30. At baseline, hypercholesterolemia and hypertension were the most common CDs with prevalences of 41% (39–43%) and 28% (26–30%) respectively. At follow-up, these prevalences increased to 64% (62–66%) and 48% (46–50%) respectively. During the study, a total of 1152 participants (62%; 60–64%) developed at least one CD while 473 (25%; 23–27%) developed at least two CDs. Among individuals without a CD at baseline, 30% (28–32%) stayed healthy (9% of the total cohort) while 70% (68–72%) developed at least one CD (21% of the cohort). Among the 1299 participants with at least one CD at baseline, 58% (56–60%) developed at least another one (41% of the cohort) while 42% (40–44%) did not (29% of the cohort).

**Table 2:** Proportion of the 1854 participants by number of CD and by individual CD status at baseline and follow-up and the number and type of CD developed during the 7.8 years of follow-up.

	CD count (n(% of column))		
	Baseline	Follow-up	New CD developed
CD count	2087	3880	1793
Number of CD			
0	555 (30)	164(9)	
1	714 (38)	496(27)	
2	428 (23)	578 (31)	
3	120 (7)	338 (18)	
4+	37(2)	278 (15)	
Multimorbidity (2+)	585 (32)	1194 (64)	
Individual CD			
Asthma	219 (12)	463 (25)	244 (13)
COPD	67 (4)	293 (16)	226 (12)
Mood and anxiety disorders	176 (10)	385 (21)	209 (11)
Other mental disorders	120 (6)	290 (16)	170 (9)
Hypercholesterolemia	756 (41)	1178 (64)	422 (23)
Diabetes	127 (7)	228 (12)	101 (5)
CVD	96 (5)	163 (9)	67 (4)
Hypertension	526 (28)	880 (48)	354 (19)

COPD: Chronic obstructive pulmonary disease and CVD: Cardiovascular disease and stroke.

### Mean age of occurrence of individual chronic diseases developed during the follow-up

**Figure 1:**
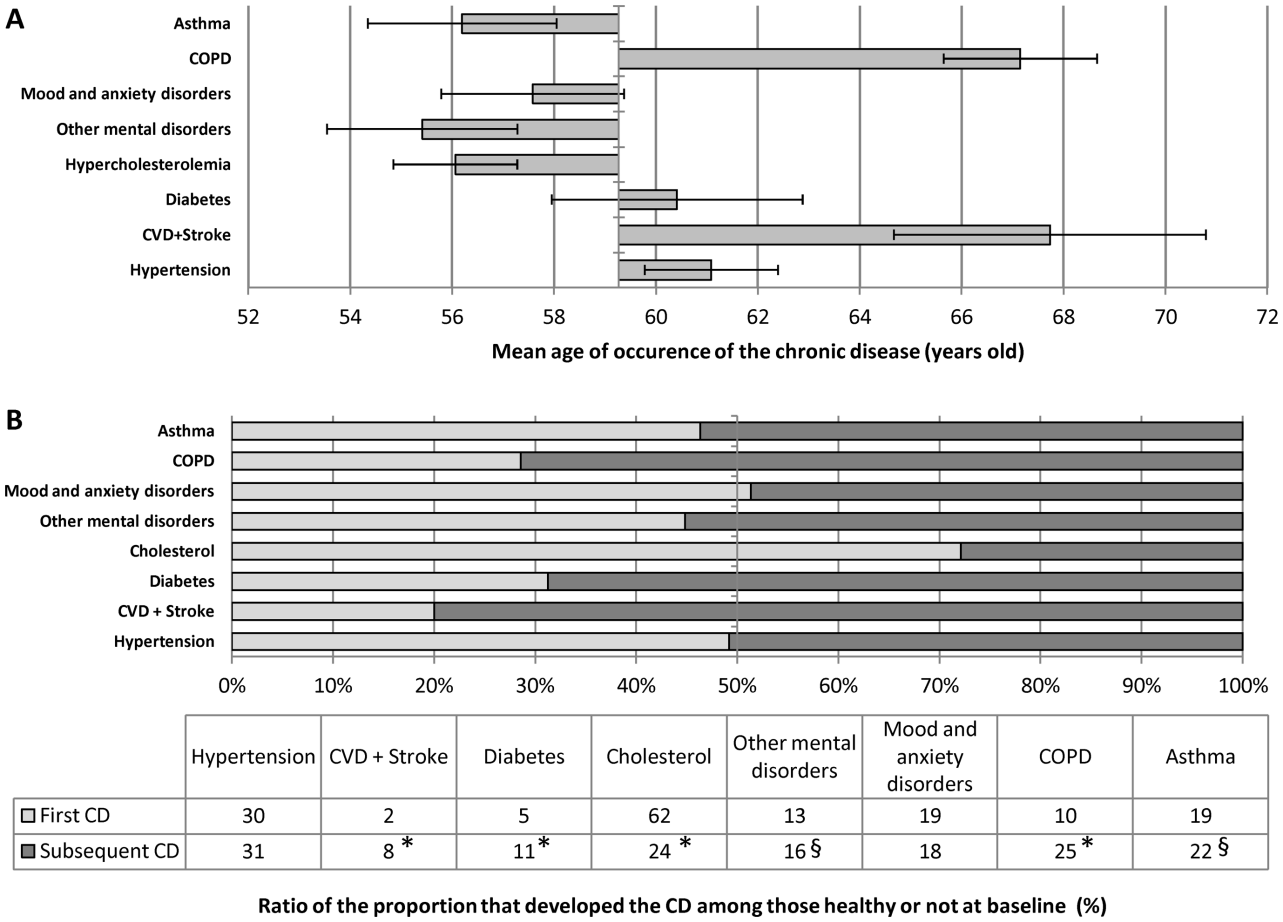
Comparison of chronic diseases developed during follow-by mean age of occurrence (Panel A), pre-existing CD status (Panel B, table) and percent occurring as a first CD (Panel B, Figure). The Y axis of Panel A correspond to the estimated mean age (59) at which CD were developed during follow-up. Error bar from panel A are confidence interval (95%). The Light and dark gray bars correspond to CD developed as a first and a subsequent CD respectively ^*^ and ^§^significantly different from the first CD group in every models and in the age and sex adjusted models respectively.

Individuals who did not develop a CD (n = 702) were significantly younger than those that developed two (n = 339) or three CDs (n = 134) during the follow-up period (mean ± S.E., −4±1, p<0.0001 and −5±1, p<0.01 years old respectively). Among the 1152 participants that developed at least one CD, the mean age of onset of all newly developed CD was 59±13 years old. The mean age of occurrence of each newly developed individual CD was determined (**Figure 1** **, panel A**). When compared to this mean age of onset of all newly developed CDs, the mean age of onset was lower for hypercholesterolemia (−4±1 years, p<0.0001), other mental disorders (−4±1 years, p<0.0001) and asthma (−3±1 years, p<0.005). Conversely, COPD (+10±1 years, p<0.0001), CVD (+10±2 years, p<0.0001) and hypertension (+3±1 years, p<0.0001) were developed at an older age.

### Comparison of baseline health status between chronic diseases developed during the follow-up

Among those who developed at least one new CD during the 7.8 years of follow-up (n = 1152), 1793 CDs were developed and 619 occurred in healthy individuals and 1174 in those who had at least one CD. The proportion of the total count of each CD developed in those who were healthy at baseline and those that had at least one CD is illustrated in **Figure 1** (**panel B**). Hypercholesterolemia represented 23% of the sum of all CDs developed. Since an individual could develop more than one CD, the proportion of the cohort that acquired the disease during the follow-up was 39%. Among those that acquired hypercholesterolima, 62% were healthy individuals at baseline while only 24% had a CD at baseline (p<0.0001). There was no significant difference in the proportion of cases that developed asthma (19% *vs*. 22%) and other mental illness (13% *vs*. 16%) between individuals without and with a CD at baseline in the crude model but this difference reached significance in both adjusted models (p<0.01 and p<0.05 respectively). The odds ratio of those with a CD at baseline over those without was 4.04 (1.92–8.51), 2.37 (1.42–3.94) and 3.01 (2.09–4.36) for CVD, diabetes and COPD new cases compared to the total number of CDs developed. Also, among those who developed at least one CD during the follow-up, 8%, 11% and 25% of the individuals with a CD at baseline acquired CVD, diabetes and COPD respectively while the proportions were only 2%, 5% and 10% for those without.

### Identification of baseline chronic diseases associated with an increased the risk to develop chronic disease during follow-up

**Figure 2:**
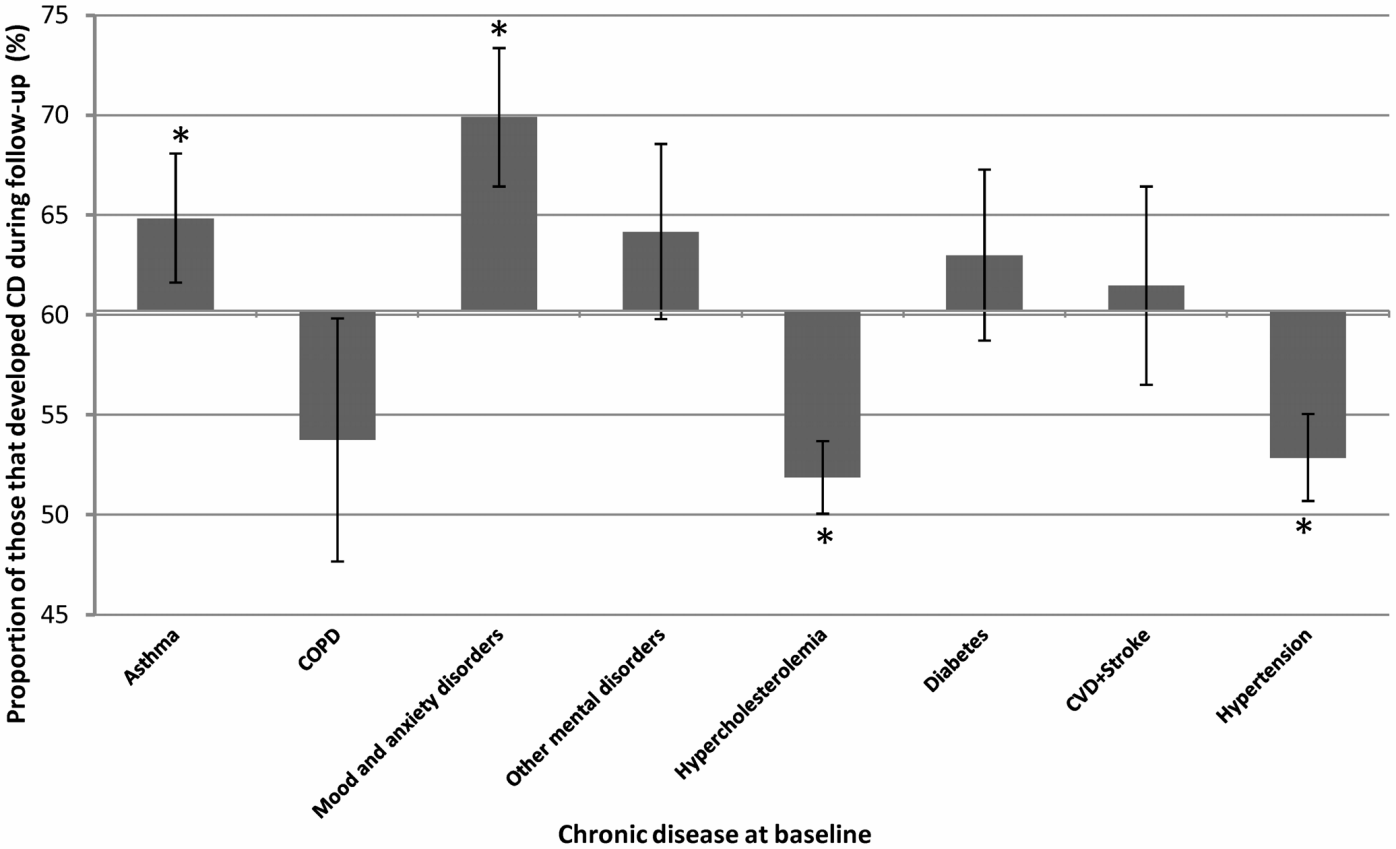
Proportion of individuals with a specific CD at baseline that developed at least one CD during the follow-up. The X-axis correspond to the proportion (60%) of those that developed at least one CD during follow-up among those that had at least one CD at baseline. *significantly different from the mean proportion of individual that developed CD. Error bar are confidence interval (95%).

Sixty percent of the population with at least one CD at baseline developed at least another one during the 7.8 year of follow-up (**Figure 2**). The proportion that developed CDs was significantly lower in those with hypercholesterolemia (52%, p<0.0001) or hypertension (53%, p<0.0001) at baseline. The proportion that developed another CD was significantly higher in those with asthma (65%, p<0.05) or mood and anxiety disorders (70%, p<0.0001) at baseline. As a consequence, the prevalence of asthma and mood and anxiety disorders at baseline were higher in those that developed a CD ((OR: 1.37 (95%CI: 1.02–1.86) and 1.76 (1.25–2.49) respectively) than in those that did not.

### Chronic diseases more likely to be developed by those with asthma or depression at baseline

Since the presence of both asthma and mood and anxiety disorders at baseline was associated with an increased risk of developing another CD, the profile of CDs that those groups developed were explored comparing them with those who had another CD at baseline and that developed another CD (**Table 3**). Individuals with asthma at baseline had a higher risk of developing COPD (OR: 1.80; 95CI: 1.22–2.67) and hypercholesterolemia (2.19; 1.48–3.25). Those having mood and anxiety disorders at baseline were more likely to develop asthma (1.62; 1.05–2.49), diabetes (2.10; 1.23–3.57) and other mental disorders (4.18; 2.69–6.49).

**Table 3:** Impact of the presence of asthma or mood and anxiety disorders at baseline on CD development as compared to those with a another CD at baseline that also developed CD.

	Asthma					Mood and anxiety disorders				
	With asthma	Without ashtma	*P*	*P* adjusted for age and sex	*P* adjusted for other risk factor^&^	With mood and anxiety disorders	Without mood and anxiety disorder	*P*	*P* adjusted for age and sex	*P* adjusted for other risk factor^&^
n	142	619				123	638			
Number of CD developed (n(%))			NS	NS	NS			<0.05	<0.05	<0.05
1	80 (56)	378 (61)				62 (50)	396 (62)*			
2	49 (34)	165 (27)				45 (37)	169 (26)*			
3+	13 (10)	76 (12)				16 (13)	73 (11)			
Individual CD developed§										
Asthma						37 (30)	134 (21)	<0.05	<0.05	= 0.06
COPD	49 (35)	140 (23)	<0.005	<0.0001	<0.001	23 (19)	166 (26)	NS	NS	NS
Mood and anxiety disorders	29 (20)	107 (17)	NS	NS	NS					
Other mental disorders	25 (18)	94 (15)	NS	NS	NS	44 (36)	75 (12)	<0.0001	<0.0001	<0.0001
Hypercholesterolemia	52 (37)	129 (21)	<0.0001	<0.0001	<0.0001	37 (30)	144 (23)	NS	NS	NS
Diabetes	13 (9)	69 (11)	NS	NS	NS	22 (18)	60 (9)	<0.01	<0.01	<0.01
CVD+Stroke	14 (10)	45 (7)	NS	NS	NS	8 (6)	51 (8)	NS	NS	NS
Hypertension	37 (26)	200 (32)	NS	NS	NS	33 (27)	204 (32)	NS	NS	NS

*significantly different between individuals with either Asthma or Mood and anxiety disorders groups and the rest of the individuals in the progressive multimorbidity groups at baseline; NS: no significant difference;

? Model adjusted for age, sex, BMI, marital status, education, income and working status at baseline,

§ The sum of this column is not 100% since some individuals developed more than one CD; CD: chronic disease; COPD: chronic obstructive pulmonary disease; and CVD =  cardiovascular disease and stroke.

### Relationship between mean age of occurrence and order of appearance of chronic diseases developed during the follow-up

**Figure 3:**
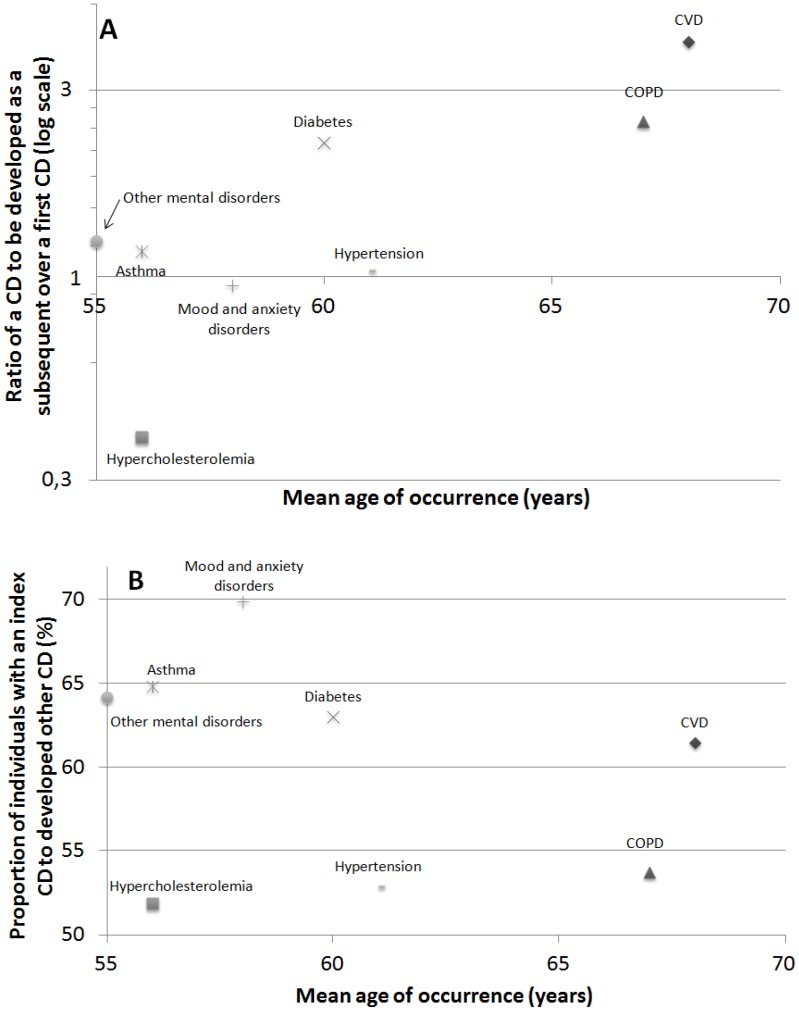
Relationship of the CD mean age of occurrence with its order of appearance (Panel A) and the risk to develop CD during the follow-up period (Panel B) and an overview of the evolution of multimorbidity (Panel C). CVD: cardiovascular disease, COPD: chronic obstructive pulmonary disease.

Relationship between mean age of occurrence of a CD and the order of appearance of a CD, expressed as the ratio of a CD to be developed as a subsequent over a first CD, is illustrated in Panel A of **Figure 3**. Briefly, hypercholesterolemia significantly occurs in younger persons and as a first CD while COPD and CVD occurs in older age groups after the occurrence of other CDs. While other mental disorders and asthma occurs at a younger age, they are as likely to occur as a first or as a subsequent CD. Diabetes is more likely to occur after the occurrence of another CD despite occurring closely to the mean age of occurrence of the eight CDs. The relationship between the mean age of occurrence and the proportion of participants with a specific CD at baseline to developing a CD during the follow-up is illustrated in Panel B of **Figure 3**. While occurring at a younger age, the proportion that developed other CDs is significantly higher among those with mood and anxiety disorder and those with asthma and lower for those with hypercholesterolemia at baseline.

## Discussion

This study contributes to the understanding of the evolution of multimorbidity using longitudinal data. Briefly, the main findings are: 1) Multimorbidity prevalence doubled over the 7.8 years of follow-up, 2) Individual CDs have different mean age of onset and order of appearance which are not always related and 3) some CDs are associated with an increased risk of developing other CDs(**Figure 3**, panel C).

### Multimorbidity and its evolution during the study

Multimorbidity is a condition affecting an important proportion of the population [2], [3], [4]. While multimorbidity has been shown to increase with age [3], [8], its longitudinal evolution is less investigated [15]. The present study reports a multimorbidity prevalence of 32% (with a mean age of 50 years old) that doubled over a mean 7.8 years of follow-up to reach 64%. This important increase in multimorbidity prevalence is in line with a recent systematic review [3], that reported that multimorbidity prevalence increases with aging, with the highest rise between age 40 and 70. Therefore, the present study, using a longitudinal design, confirms the steep and important increased in the prevalence of multimorbidity with aging reported in cross sectional studies. Also, the proportion of those with at least one CD was found to be similar to those observed in general practitioner populations but higher than those estimated in the general population [5].

### Mean age of onset of chronic diseases developed during follow-up

This study also presents differences in the mean age of onset for eight CDs in the same population. Briefly, the mean age of onset of asthma, hypercholesterolemia and other mental disorders is younger while COPD, CVD and hypertension is older. While mental disorders are known to occur at a young age [27], it is surprising that mood and anxiety disorders are not frequent in the younger age group, particularly considering that their respective mean age of onset has been previously estimated to be 11 years for anxiety and 30 years for mood disorders [27]. The reported mean age of onset of diabetes, asthma and COPD are in line with previous publications [28], [29]. The present observations result from incident cases from individuals over 18 years old and do not take into account the age of onset of CDs already present at baseline. Despite these limits, the mean age of onset is important in understanding the evolution of multimorbidity and can be used to compare factors associated with CD occurrence. To the best of our knowledge, the comparison of age of onset for multiple CDs with different aetiology has never been undertaken.

### Integration of order of appearance and mean age of occurrence for different chronic diseases

Hypercholesterolemia is more likely to occur at a younger age and as a first CD. This is concordant with a previous study showing that this CD was not associated with multimorbidity in populations under 60 years old [4] and the role of the hypercholesterolemia in diabetes and CVD development [30]. COPD and CVD had an older mean age of occurrence and were more than twice as likely to develop after other CDs. Conversely, hypertension, which also had an older mean age of occurrence, is not more likely to occur as a subsequent CD than as a first. Asthma and other mental illness, which occurs at a younger age, were more likely to develop in those with a previous CD in both models adjusted for age and sex. Briefly, hypercholesterolemia, mood and anxiety disorders, COPD and CVD have a corresponding mean age of occurrence and order of appearance but not the other CDs.

### Hypertension and hypercholesterolemia decrease the risk of developing chronic diseases

Another finding of the present study is that the presence of hypertension and hypercholesterolemia at baseline was significantly associated with a lower risk of developing CDs. This could be explained, at least partly, by the fact that statins and blood pressure lowering drugs are known to delay the development of some CDs such as CVD and diabetes [30]. While hypertension and hypercholesterolemia were associated with a decreased risk of developing other CDs, their high prevalence still makes them the most common CD developed in individuals with existing CDs.

### Mood and anxiety disorders increase the risk of developing diabetes, asthma and other mental disorders

The presence of mood and anxiety disorders or asthma at baseline was associated with an increased risk of developing other CDs. Over a third of the individuals with a mood and anxiety disorder develop another mental health problem over 7.8 years. It is well known that mood and anxiety disorders could trigger numerous other mental conditions [31], [32]. Mood and anxiety disorders are also associated with multiple physical conditions such as diabetes, CVD, cancer and asthma [33], [34], [35], [36], [37]. In the present cohort, 18% of the individuals with mood and anxiety disorders developed diabetes over the 7.8 years of follow-up. This increase is twice the one observed in other individuals who developed at least another CD. Mood and anxiety disorders had previously been associated with diabetes [34], [35] and its development [38]. Among the mechanisms proposed to explain this association, the use of antidepressants, particularly tricyclics antidepressants and selective serotonin reuptake inhibitors, in individuals with mood and anxiety disorders has been associated with an increased risk of developing abdominal obesity and diabetes [38], [39], [40]. While some work has been done to determine the impact of mood and anxiety disorders and its treatment on comorbidities [41], [42], not much has been undertaken to determine their actual impact on other CD development. In light of the present results showing an increased risk in individuals with mood and anxiety disorders to develop diabetes, other mental disorders and asthma, as well as its consequences on other CD management and related complications [43], [44], this study supports the development of new approaches to address this problem [41].

### Asthma increases the risk of developing COPD and hypercholesterolemia

In the present study, those with asthma at baseline were more likely to develop COPD and hypercholesterolemia. While asthma has a younger age of onset, it is also associated with higher comorbidity rates [45]. In the present study, over 30% of those that developed more than two CDs developed both hypercholesterolemia and asthma which suggest an association between those two CDs (data not shown). In support of this hypothesis, asthma has been related to a disorder in cholesterol metabolism [46], [47]. However, much work needs to be done to better understand and support this hypothesis. During the 7.8 years of follow-up, over 35% of the individuals with asthma developed COPD. This is in line with a recently reported overlapping in both asthma and COPD that could be associated with more frequent and severe respiratory exacerbations despite a younger age and a reduced lifetime smoking history. In the present study, the younger age observed in this population supports this observation [48], [49], [50].

### Limits

While data have been adjusted for risk factors such as age, sex, education, marital status, working status, annual income and BMI, some potential residual confounders, such as nutrition and sedentary lifestyle, could still have an impact on multimorbidity evolution [16]. The number of CDs investigated in the present study (8) is also relatively small when compared to some multimorbidity studies [3]. However, the diagnosis of most of those CDs is based on biomedical measurement which is more precise than most other data sources since it ensures consistency of measurement despite potential change in CD definition, identification and screening procedure. It also permits taking into account undiagnosed conditions which has been previously shown to be high for some CDs [51]. In the present study, those having a CD were considered as having it for every phase afterward which could have the potential to include milder forms of CD such as milder hypertension which could be considered as solved with appropriate lifestyle modifications. Also, individuals that died during the follow-up are not included and this may bring an immortal bias. However, this study is focusing on the evolution of multimorbidity and not on the impact of CDs on mortality. The quality of the diagnosis, the 7.8 years longitudinal follow-up and the use of adapted control group are among the strengths of the study.

## Conclusions

In summary, this study presents some of the first observations on the evolution of multimorbidity. This approach shows the importance of the increase in multimorbidity prevalence, that CDs occur at different ages, in different order of appearance and that their occurrence could have an impact on the development of other CDs.
